# THE EFFECT OF PANDEMICS ON TRUST IN DOCTOR IMPROVEMENT AMONG CHINESE OLDER ADULTS: LESSONS FROM COVID-19

**DOI:** 10.1093/geroni/igad104.3635

**Published:** 2023-12-21

**Authors:** Chao Guo, Xiyuan Hu, Dianqi Yuan, Huameng Tang, Peisen Yang

**Affiliations:** Peking University, Beijing, Beijing, China (People’s Republic); Peking University, Beijing, Beijing, China (People’s Republic); Peking University, Beijing, Beijing, China (People’s Republic); Peking University, Beijing, Beijing, China (People’s Republic); Peking University, Beijing, Beijing, China (People’s Republic)

## Abstract

The COVID-19 pandemic has had a major impact on the physician-patient relationship. For older adults, trust in doctors during the pandemic affects their lives and health. This study aimed to estimate the effect of the first wave of COVID-19 transmissibility in China on older adults’ trust in doctors. We obtained individual data from the Chinese Family Panel Studies (CFPS). A total of 2,225 participants aged 65 and older were included in this study. The COVID-19 pandemic was used as a natural experiment. We obtained difference-in-differences (DID) estimates of the pandemic’s effects by exploiting temporal variation in the timing of COVID-19 exposure across participants interviewed from 2018 to 2020 together with the geographical variation in COVID-19 severity at the provincial district level. Overall, the DID estimates showed that the COVID-19 pandemic could significantly increase trust in doctors after controlling for multiple covariates (adjusted β: 0.053, 95% CI: 0.028–0.078). Stratified analyses showed that the effect was only in females and the more educated ones but not in their counterparts. In addition, the COVID-19 pandemic showed a positive effect on trust in doctors among 65-79-year-olds (0.082, 0.056–0.109), but older adults over the age of 80 showed significantly reduced trust in doctors (-0.236, -0.418–-0.055). Our findings confirm the association between the COVID-19 pandemic and trust in doctors among older Chinese people. Hospitals and other medical organizations should pay more attention to improving older adults’ trust in doctors and building a better physician-patient relationship to face the future pandemic.

